# Intestinal Production of Anti-Tissue Transglutaminase 2 Antibodies in Patients with Diagnosis Other Than Celiac Disease

**DOI:** 10.3390/nu9101050

**Published:** 2017-09-21

**Authors:** Mariantonia Maglio, Fabiana Ziberna, Rosita Aitoro, Valentina Discepolo, Giuliana Lania, Virginia Bassi, Erasmo Miele, Tarcisio Not, Riccardo Troncone, Renata Auricchio

**Affiliations:** 1 European Laboratory for the Investigation of Food Induced Disease (ELFID), University Federico II, 80131 Naples, Italy; mariantonia.maglio@unina.it (M.M.); giuliana.lania@gmail.com (G.L.); r.auricchio@unina.it (R.A.); 2 Institute for Maternal and Child Health—IRCCS “Burlo Garofolo” Trieste, 34137 Trieste, Italy; fabyz1@alice.it (F.Z.); tarcisio.not@burlo.trieste.it (T.N.); 3 Department of Medical Translational Science, Section of Paediatrics, University Federico II, 80131 Naples, Italy; aitoro.rosita@gmail.com (R.A.); vale.discepolo@gmail.com (V.D.); virginiabassi86@gmail.com (V.B.); erasmo.miele@unina.it (E.M.); 4 Department of Reproductive, Developmental and Public Health Sciences, University of Trieste, 34137 Trieste, Italy

**Keywords:** celiac disease, potential celiac disease, intestinal anti-transglutaminase 2 antibodies, TCR-γδ^+^ intraepithelial lymphocytes

## Abstract

It has been hypothesized that gluten-dependent production of anti-tissue-transglutaminase 2 (anti-TG2) antibodies may occur only at an intestinal level. We have investigated intestinal production of anti-TG2 antibodies in 136 patients with normal serum levels of anti-TG2 antibodies and normal duodenal mucosa. Intestinal deposits of anti-TG2 antibodies were evaluated by immunofluorescence and anti-TG2 antibodies released in organ culture supernatants measured by ELISA. Intestinal antibody libraries were obtained from 10 subjects. Immunohistochemistry for CD25^+^, CD3^+^, and TCR-γδ^+^ was assessed in subjects with positive (*n* = 32) and negative (*n* = 31) intestinal anti-TG2 antibodies. Globally 33/136 (24%) seronegative patients produced anti-TG2 autoantibodies at an intestinal level. Antibody libraries analysis confirmed the anti-TG2 antibodies mucosal production in all (*n* = 8) positive subjects. Lamina propria CD25^+^ cell count was significantly (*p* < 0.05) higher in patients with intestinal anti-TG2. Moreover, 13/32 (41%) of them showed high TCR-γδ^+^/CD3^+^ ratios. Intestinal anti-TG2 antibody production does not show absolute specificity for CD. It is seen more often in association with inflamed mucosa. Further investigations are necessary to prove the possible role of dietary gluten.

## 1. Introduction

Celiac disease (CD) is considered a systemic immune-mediated disorder elicited by gluten and related prolamines in genetically susceptible individuals [1]. The presence in the serum of anti-tissue-transglutaminase 2 (anti-TG2) antibodies is considered a sensitive and specific biomarker for CD [2].

Anti-TG2 antibodies are produced primarily at an intestinal level by specific B lymphocytes and, using phage display library technology, it has been proven that these antibodies are synthesized in CD patients with preferential use of IGVH5-51 gene family [3]. Once produced, anti-TG2 antibodies can deposit in the small intestinal mucosa [4], even before they can be detected in the circulation [5,6]. Furthermore, TG2 specific IgA deposition precedes the formation of small intestinal lesion in CD [6,7] as it can be detected also in potential CD patients [6,8,9], i.e., patients showing positive serology for CD-specific antibodies, but architecturally normal intestinal mucosa [10]. The presence of mucosal deposits of anti-TG2 antibodies seems to be a predictive marker of future evolution to villous atrophy, as it was seen more often in those potential patients that progress to overt CD than in those remaining in early phase of disease [5,6,8].

Several investigators have shown that intestinal anti-TG2 IgA deposits are also detectable in subjects with diagnosis other than CD with a prevalence ranging from 5 to 20% [11,12,13]; in our experience, up to 12% of pediatric non-CD patients shows mucosal deposits [13]. A similar percentage of non-CD patients producing anti-TG2 antibodies only at an intestinal level has been recently shown by us by measuring anti-TG2 antibodies secreted into supernatants from organ culture of small intestinal biopsy [9]. This phenomenon could be not specific for CD and merely attributable to intestinal inflammation; alternatively, it could be suggestive of a very early condition of gluten reactivity. The subjects initially producing anti-TG2 antibodies only at an intestinal level could become serum positive later and eventually develop small intestinal mucosal damage.

Here we aimed to investigate the intestinal production of anti-TG2 antibodies in patients with normal serum levels of such antibodies and to relate that production to immunohistochemical markers of gluten-dependent enteropathy.

## 2. Materials and Methods

### 2.1. Patients

In this retrospective study, we considered 283 patients enrolled between January 2001 and December 2015 at the Department of Pediatrics of the University Federico II in Naples, for whom samples of small intestinal biopsies fixed in formalin, biopsies frozen in optimal cutting temperature compound and organ culture supernatants were available. One-hundred and forty-seven of 283 patients (40 males, mean age 6.9 years) had high serum levels of anti-TG2 antibodies and/or positive serum anti-endomysium antibodies, they underwent a small intestinal biopsy for suspicion of CD. Forty-four of 147 showed a lesion of intestinal mucosa type Marsh 3, according to Marsh classification modified by Oberhuber et al. [14], and had a final diagnosis of CD according to ESPGHAN criteria (1990, 2012) [1,15]; they were referred to as Active CD. One-hundred and three of 147 patients had a normal or slightly infiltrated duodenal mucosa classified as Marsh 0 (*n* = 40) and Marsh 1 (*n* = 63). They were defined as Potential CD. One-hundred and thirty-six out of 283 subjects (66 males, mean age 7 years) had normal values of anti-TG2 antibodies and/or absence of anti-endomysium antibodies in the serum. We found that 130/136 showed duodenal mucosa Marsh 0 *(n* = 98) or Marsh 1 (*n* = 32), and 6/136 showed Marsh 3a mucosa. They had final diagnosis other than CD: gastroesophageal reflux (*n* = 52), gastrointestinal functional disorders (*n* = 32), type 1 diabetes (*n* = 19), *Helicobacter pylori* infection (HP, *n* = 10), eosinophilic esophagitis (*n* = 8), first degree relatives of CD patients (*n* = 6), iron deficiency anemia (*n* = 2), failure to thrive (*n* = 2), inflammatory bowel disease (*n* = 2), and autoimmune hepatitis (*n* = 2). They represented the Non-CD group.

IgA deficiency was ruled out in all enrolled subjects. The use of biopsy specimens was authorized by the Institutional Ethical Committee.

### 2.2. Detection of Mucosal Deposits of anti-TG2 IgA Antibodies

The presence of intestinal deposits of anti-TG2 IgA was investigated on duodenal frozen sections from all patients. Five µm sections were stained using a double-immunofluorescence method, as previously described [16]. The stained sections were evaluated using a fluorescent microscope (Axioskop2 plus; Zeiss MicroImaging Inc., Milan, Italy).

### 2.3. Biopsy Specimens and Organ Culture

During upper gastrointestinal endoscopy, at least four duodenal biopsies were taken from all patients and fixed in 10% formalin, embedded in paraffin, and then treated for histological and morphometrical analysis performed by light microscopy and by two experienced pathologists. A villous height crypt depth ratio ≥2.2 was considered normal [17]. One further duodenal specimen was embedded in an optimal cutting temperature compound (OCT; Killik, Bio-Optica, Milan, Italy) and stored in liquid nitrogen until used. The last fragment was placed on a stainless steel mesh positioned over the central well of an organ culture dish with the villous surface of the specimens upper-most in medium containing RPMI 1640 (80%; Sigma, Milan, Italy) supplemented with fetal bovine serum (15%; Life Technologies-GibcoBRL, Milan, Italy), l-glutamina (2 mM; Life Technologies-GibcoBRL), penicillin (100 U/mL), streptomycin (100 μg/mL) (Life Technologies-GibcoBRL), and insulin (1 mg/mL; Sigma). The biopsy was cultured for 24 h at 37 °C with the culture medium; it was placed in a sterile anaerobic jar which was gassed with 95% oxygen/5% carbon dioxide. The supernatants were collected and stored at −80 °C until they were analyzed.

### 2.4. Measurement of Anti-TG2 IgA Antibodies Secreted into Culture Supernatants

Mucosal anti-TG2 IgA antibodies secreted into culture supernatants were measured in undiluted supernatants by enzyme-linked immunosorbent assay (ELISA; EU-tTG IgA kit; Eurospital S.p.A., Trieste, Italy), according to the manufacturer’s instructions. The cut-off value was previously calculated as 2.8 U/mL [9]. When the value of anti-TG2 was higher than the last point of standard curve, supernatants were diluted 1:20 in culture medium.

### 2.5. Phage Display Library

Total tissue RNA was extracted from whole duodenal biopsies using TRIZOL reagent (Gibco Life Technologies, Milan, Italy). cDNAs were generated from total RNA using the High-Capacity cDNA Reverse Transcription Kit (Applied Biosystems, Waltham, MA, USA). Selective IgA IGVH5-51 genes were amplified from cDNA and assembled into single chain fragment-variable (scFv) fragments by cloning into phagemid vector pDAN5 as previously reported [18]. After selection, by affinity chromatography, 45 individual clones were screened for reactivity to TG2 by ELISA. To value their diversity, clones were sequenced. The VH family and the V gene were analyzed using IMGT V-quest database [19].

### 2.6. Immunohistochemistry

Immunohistochemical staining for CD3^+^, TCR-γδ^+^, and CD25^+^ cells was performed using four-µm frozen duodenal sections as previously reported [20]. Briefly, duodenal cryostat sections were fixed in acetone for 10 min. After incubation with normal rabbit serum (1:100, Dako, Copenhagen, Denmark) for 20 min, sections were covered with anti-CD3 (1:100; Dako), anti-TCRγδ (1:60; Thema Ricerca, Castenaso (BO), Italy), or anti-CD25 (1:20; Exalpha biological Inc., Shirley, MA, USA), monoclonal antibodies for 1 h. The sections treated for detection of CD3^+^ and TCR-γδ^+^ cells were then covered with anti-mouse Envision system-HRP (Dako) for 45 min. The sections treated for detection of CD25^+^ cells were then covered with rabbit anti-mouse immunoglobulins for 30 min and successively with monoclonal mouse alkaline phosphatase anti-alkaline phosphatase (APAAP; 1:40; Sigma-Aldrich, Milan, Italy) for 30 min. 2-amino-9-ethyl-carbazole (AEC) (Sigma-Aldrich, Milan, Italy) and new fuchsine were used as peroxidase and phosphatase alkaline substrate, respectively. Finally, sections were counterstained with Mayer’s hematoxylin (Sigma-Aldrich) and mounted with Aquamount (BDH, Poole, England). Monoclonal antibodies were diluted in Tris pH 7.4. All incubations were conducted at room temperature in a humid chamber. As a negative control, mouse IgG2a/IgG1 (Dako) replaced the primary antibody.

The number of stained cells per millimeter of epithelium determined the density of cells expressing CD3 and TCR-γδ in the intraepithelial compartment. Cut-off values for CD3^+^ and TCR-γδ^+^ cells were 34/mm and 3.4/mm of epithelium, respectively. On the other hand, the number of cells expressing CD25 in the lamina propria was evaluated within a total area of 1 mm^2^. The cut-off value for CD25^+^ cells was 4/mm^2^ lamina propria. Morphometric evaluations were performed using a microscope (Axioscop, Zeiss MicroImaging Inc., Milan, Italy) with a calibrated lens aligned parallel to the muscolaris mucosae.

### 2.7. Statistics

Statistical analysis was performed using GraphPad Prism 4 for Windows, version 4.03. Data were compared by Mann–Whitney test and Fisher’s χ^2^ test. A *p*-value < 0.05 was considered to be significant.

### 2.8. Ethical Approval

Written informed consent was obtained from parents of children enrolled. The study protocol was approved by the Ethical Committee of the University ‘Federico II’ Naples, Italy (CE 230/05).

## 3. Results

### 3.1. Intestinal Anti-TG2 Antibodies Are Also Produced in Non-CD Patients

Mucosal deposits of IgA anti-TG2 antibodies were detected in 40 of 44 Active CD (90.9%), 72 of 103 Potential CD (69.6%), and 25 of 136 Non-CD patients (18.3%). In CD patients, they presented as thick bands under surface epithelium, around crypts and vessels. In potential CD subjects the bands were less thick with a patchy distribution. In patients with diagnosis other than CD, the staining appeared weak, in some cases deposits were evident only around some crypts.

On the other hand, in all CD patients in the active phase of disease (100%), in 101/103 (98%) patients with potential CD and in 13/136 subjects (10%) with diagnosis other than CD, anti-TG2 antibodies could be measured in culture supernatants. Anti-TG2 antibody titers ranged from 4.21 to 2020.3 U/mL in CD and from 3.3 to 29.3 U/mL in Non-CD patients.

Our data showed that in total 33/136 Non-CD patients (24.3%) had evidence of intestinal production of anti-TG2 antibodies: they were defined below as Positive Non-CD patients. Five of 136 Non-CD subjects were found positive for both assays.

The 33 Positive Non-CD patients had following diagnoses: type 1 diabetes (*n* = 10), gastroesophageal reflux (*n* = 9), gastrointestinal functional disorders (*n* = 6), first degree relatives of CD patients (*n* = 3), eosinophilic esophagitis (*n* = 3), HP infection (*n* = 2). 29/33 patients had Marsh 0 (*n* = 24) or Marsh 1 (*n* = 5) duodenal mucosa and the remaining 4 patients had a Marsh 3a mucosa.

All CD patients had HLA-DQ2 and/or DQ8 haplotypes. The celiac disease-type HLA was known in 14/33 Positive Non-CD and in 5/31 Negative Non-CD patients. It was found that 12/13 Positive Non-CD were HLA-DQ2 and/or -DQ8 positive, 2 patients showed DQA1*05-DQB1*03 haplotype, the most frequent HLA haplotype in DQ2/DQ8 negative CD patients [21]. Three of the five Negative Non-CD patients were HLA-DQ2 positive, the last two were HLA-DQ2/DQ8 negative.

### 3.2. Phage Display Technology Confirms the Intestinal Production of Anti-TG2 Antibodies in Non-CD Patients

To confirm the intestinal production of anti-TG2 antibodies in positive Non-CD patients, we created IGVH5-51 antibody libraries against TG2 from biopsy specimens of 8/33 Positive Non-CD patients (six positive to detection of mucosal deposits of anti-TG2 antibodies and two who secreted anti-TG2 into culture supernatants); two Non-CD patients negative to both the above mentioned tests served as controls (Negative Non-CD).

Antibody clones recognizing TG2 were isolated from intestinal biopsies of all Positive Non-CD patients. The number of anti-TG2 antibody clones ranged from 2.2% to 13.3%. No difference in percentage of anti-TG2 specific clones was observed between the patients positive only to detection of mucosal deposits and the patients positive only to measurement of anti-TG2 antibodies into culture supernatants. No anti-TG2 antibody clones were isolated from biopsy samples of the two patients double negative to the above mentioned tests (Table 1). All positive Non-CD patients, with the exception of the two affected by type 1 diabetes, produced anti-TG2 antibodies with a preferential use of VH5 gene family. The type 1 diabetes patients produced anti-TG2 antibodies mainly belonging to the VH3 gene family.

### 3.3. Positive Non-CD Patients Presented Signs of Activated Cell-Mediated Mucosal Immunity

In order to assess the activation of mucosal immunity and in particular of a gluten-related deranged immune response, the density of mononuclear cells expressing CD25 in *lamina propria* and the density of CD3^+^ and TCR-γδ^+^ cells in epithelial compartment were investigated in 32/33 Positive Non-CD patients and in 31/103 Non-CD patients negative to both above-mentioned tests (Negative Non-CD) that served as control group. Twelve of 32 (37%) Positive Non-CD and 5/31 (16%) Negative Non-CD presented with alteration of two or more of the above-mentioned markers, (Table 2).

**Figure 1:**
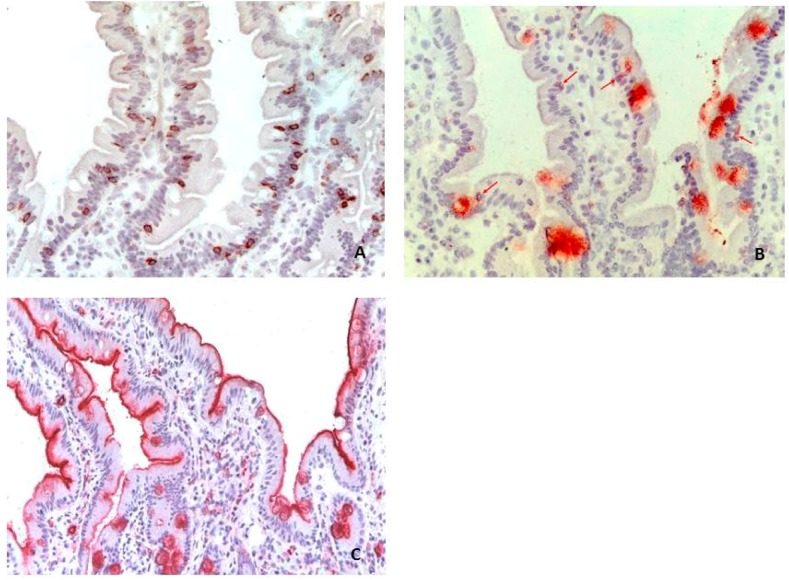
Immunohistochemical stained cells in duodenal sample from a Positive Non-CD patient. (**A**) CD3^+^ intraepithelial lymphocytes (IELs) (brown cells); (**B**) TCR-γδ^+^ IELs (red arrows) infiltrating surface epithelium; and (**C**) CD25^+^ lamina propria mononuclear cells (fuchsia cells) in Marsh 0 duodenal sample of one Positive Non-CD patient. 200× magnification.

**Figure 2:**
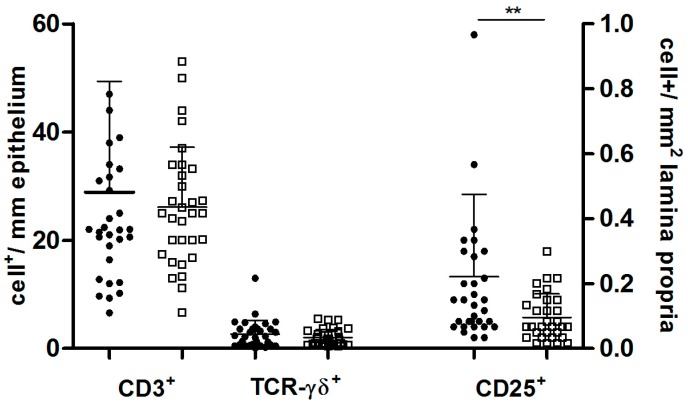
Immunohistochemical markers of immune activation in the epithelium and lamina propria of Non-CD patients. Mononuclear CD25^+^ cells are increased in Positive Non-CD patients. Each point represents a single patients. The black points represent Non-CD patients producing anti-TG2 antibodies at intestinal level; the white squares represent Negative Non-CD patients. Horizontal bars indicate median values. Mann–Whitney test, ** *p* < 0.01.

In the Positive Non-CD group, 7 of 32 patients (21.8%) presented increased number of CD3^+^ intraepithelial lymphocytes (IELs) (mean ± SD = 28.9 ± 20.5 cells) (Figure 1A), 11 of 32 patients (34.3%) increased number of TCR-γδ^+^ IELs (mean ± SD = 2.6 ± 2.5 cells) (Figure 1B) and 24 of 32 (75.0%) increased number of *lamina propria* CD25^+^ mononuclear cells (mean ± SD = 13.2. ± 15.2 cells) (Figure 1C). On the contrary in the Negative Non-CD group, high number of CD3^+^ (26.1 ± 11.0), TCR-γδ^+^ (2.0 ± 1.5) and CD25^+^ (5.7 ± 4.3) cells were found in 5/31 (16.1%), 6/31 (19.3%) and 14/31 (45.1%) patients, respectively (Table 2). Only the percentage of subjects with an increased number of CD25^+^ mononuclear cells in lamina propria was significantly higher in Positive Non-CD in comparison to Negative Non-CD patients (*p* < 0.05) (Table 2). Likewise the density of CD25^+^ cells in lamina propria was significantly higher in Positive Non-CD (13.2 ± 15.12 cells) than that in Negative Non-CD (5.7 ± 4.31 cells) (*p* < 0.01) (Figure 2). Finally, we evaluated TCR-γδ^+^/CD3^+^ ratio that is considered a more reliable marker of gluten sensitivity than CD3 and TCR-γδ values considered singularly [20]. 41% (13/32) of Positive Non-CD showed TCR-γδ^+^/CD3^+^ ratio over cut-off in comparison to 29% (9/31) of Negative Non-CD (Table 2). The diagnosis of those with high TCR-γδ^+^/CD3^+^ ratio were: gastroesophageal reflux (*n* = 6), type 1 diabetes (*n* = 4), eosinophilic esophagitis (*n* = 2), gastrointestinal functional disorders (*n* = 1).

## 4. Discussion

In this retrospective study we showed that the great majority of active CD patients produce anti-TG2 antibodies at the level of the intestinal mucosa. In our laboratories, two tests were used to detect intestinal anti-TG2 antibodies: the first is based on the co-localization of antibodies against TG2 and their natural tissue antigen in duodenal cryostat sections by immunofluorescence [4,16]; the second one is an ELISA test performed on supernatants from duodenal biopsy culture [9] and, in this pediatric population, it has a higher sensitivity compared to deposits detection, confirming our previous data [9,13]. Moreover, ELISA test detects spontaneous intestinal production of CD-specific autoantibodies even in the very early stage of the disease. Indeed, it results positive in 98% of potential CD, whose intestinal mucosa does not show signs of damage and in whom serum levels of anti-TG2 antibodies are lower than those found in the active phase of disease [22]. Of note, mucosal deposits are detectable only in about 70% of pediatric patients with potential CD, thus suggesting that even very low antibody production may be detected by ELISA testing in culture supernatants. The absence of mucosal deposits of anti-TG2 antibodies, more often noted in potential CD, could be due to the low affinity displayed by these antibodies to their antigen and/or to their lower concentration.

A highly sensitive test able to demonstrate the intestinal production of anti-TG2 antibodies could be relevant in clinical practice for its possible ability to predict evolution towards mucosal damage. Moreover, the ability to detect a very early production of intestinal anti-TG2 antibodies could be helpful to identify a condition of gluten sensitivity in patients with absence of serum CD-associated autoantibodies [23,24].

On the other hand, we showed that the above-mentioned tests have a high specificity even though they do not reach 100%. In this particular population, approximately 25% of Non-CD patients produce anti-TG2 antibodies only at intestinal level. Our data showed that some of those patients without circulating anti-TG2 antibodies exhibit mucosal deposits of anti-TG2 antibodies with a pattern similar to that observed in potential CD and/or spontaneously secrete the same antibodies in supernatants after 24 hours of small intestine culture. The results obtained using the phage display technologies definitely support the above-mentioned data. Indeed, the construction of antibody libraries from biopsy samples showed a production of an anti-TG2 specific antibody repertoire dominated by the usage of VH5-51 gene segment (in 75% of patients), the same used by active CD patients [3,25], and by the VH3 (in 25%) gene segment. Of interest, the only two first degree relatives of CD patients included in the phage display library analysis of this study showed a VH5-51 gene usage, confirming a previous report by Not et al. [24]. Furthermore, two of the patients studied by phage display library have type 1 diabetes and mainly produce VH3 anti-TG2 antibodies, as we already previously published [26]. With the same technology we were not able to isolate TG2-specific antibody clones from Non-CD patients who did not present any sign of intestinal anti-TG2 antibody production. Altogether, our data showed an intestinal production of anti-TG2 antibodies in a subgroup of Non-CD patients.

The demonstration of intestinal production of anti-TG2 antibodies in non-CD patients, poses the question of whether this finding is related to a deranged mucosal immune response to gluten, as shown in other settings, such as in first-degree relatives of CD patients [24] or in patients with genetic gluten intolerance [24,27]. Notably, individuals producing intestinal anti-TG2 antibodies, taken as a group, more often (75% of them) show inflammatory signs, demonstrated by the increased density of CD25^+^ cells, in the small intestinal mucosa as compared to Non-CD subjects lacking evidence of local anti-TG2 antibodies production. Furthermore, they tend to present increased density of markers considered specific for gluten-sensitive enteropathy, such as TCR-γδ^+^ intraepithelial lymphocytes [28] and increased value of TCR-γδ^+^/CD3^+^ ratio [20]. On the other hand, it is known that the increase of intraepithelial lymphocytes, peculiar of Marsh 1 duodenal lesion, and also the higher intraepithelial density of TCR-γδ^+^ lymphocytes have no absolute specificity for CD [29,30].

Our findings are not surprising for subjects with type 1 diabetes and first-degree relatives of CD patients included in our study group, since they belong to groups at risk to develop CD [28,31], but are intriguing for individuals unrelated to CD. However, the association between gastrointestinal disorders such as eosinophilic oesophagitis, gastro-oesophageal reflux disease, or *Helicobacter pylori* infection and intestinal production of anti-TG2 antibodies is to date unknown as yet little explored.

HLA typing could be of help as the absence of HLA-DQ2/-DQ8 alleles in these patients would suggest the hypothesis that the production of anti-TG2 antibodies at intestinal level is not exclusive of CD. Although we have typed for HLA only 42% of intestinal anti-TG2 positive Non-CD patients, all cases but two turned out to be HLA-DQ2/-DQ8 positive and the two negative cases have DQA1*05-DQB1*03 haplotype, the most frequent HLA haplotype in DQ2/DQ8 negative CD patients [21].

Both possibilities remain then open: intestinal anti-TG2 IgA production could be due to a general state of inflammation or could indicate a very early stage of a gluten-dependent pathology. The effect of a gluten free diet and a more prolonged clinical follow-up could help to establish whether our finding is expression of a gluten-dependent mucosal response and if those subjects belong to the spectrum of gluten-related disorders.

## Figures and Tables

**Table 1:** Laboratory data of the intestinal immune anti-TG2 response from the 10 selected Non-CD Patients

Patient	Deposits Anti-TG2	Anti-TG2 into Supernatants	Anti-TG2 VH	HLA
*n* = 1 Non-CD	Neg	+	VH5	ND
*n* = 2 Non-CD	Neg	+	VH5	ND
*n* = 3 Non-CD	+	Neg	VH5	ND
*n* = 4 Non-CD	+	Neg	VH5	DQ2/DQ8 negative (DQ7DR5)
*n* = 5 Non-CD	+	Neg	VH5	ND
*n* = 6 Non-CD	Neg	Neg	No	DQ2 positive
*n* = 7 Non-CD	+	Neg	VH5, VH3	DQ2/DQ8 positive
*n* = 8 Non-CD	+	Neg	VH5, VH3	DQ2/DQ8 positive
*n* = 9 Non-CD	Neg	Neg	No	DQ2 positive
*n* = 10 Non-CD	Neg	Neg	No	DQ2 positive

Anti-TG2 = anti-tissue transglutaminase2 antibodies; ND = not determined.

**Table 2:** Immunohistochemical markers of gluten-related enteropathy tested in 32 Positive Non-CD patients and in 31 Negative Non-CD patients.

Marker	Positive No-CD Patients	Negative No-CD Patients	Χ^2^ Fisher’s Exact Test
CD25^+^ cells > cutoff	24/32 (75.0%)	14/31 (54.1%)	*p* = 0.02
CD3^+^ cells > cutoff	7/32 (21.8%)	5/31 (16.0%)	*p* = 0.5
TCR-γδ^+^ cells > cutoff	11/32 (34.4%)	6/31 (19.3%)	*p* = 0.2
TCR-γδ^+^/CD3^+^ ratio > cutoff	13/32 (40.6%)	9/31 (29.0%)	*p* = 0.4
